# MTDH promotes metastasis of clear cell renal cell carcinoma by activating SND1-mediated ERK signaling and epithelial-mesenchymal transition

**DOI:** 10.18632/aging.102694

**Published:** 2020-01-24

**Authors:** Anbang He, Shiming He, Cong Huang, Zhicong Chen, Yucai Wu, Yanqing Gong, Xuesong Li, Liqun Zhou

**Affiliations:** 1 Department of Urology, Peking University First Hospital, Beijing 100034, China; 2 Institute of Urology, Peking University, Beijing 100034, China; 3 National Urological Cancer Center, Beijing 100034, China

**Keywords:** clear cell renal cell carcinoma, metastasis, MTDH, SND1, EMT, ERK

## Abstract

Background: Metastasis is the principal cause of renal cell carcinoma-associated mortality. Metadherin (MTDH) was identified as a vital metastasis driver involved in the metastatic progression of various types of tumors, suggesting that MTDH is a prognostic metastatic biomarker and potential therapeutic target. The role and mechanism of MTDH in the metastatic progression of ccRCC have not yet been adequately explored.

Results: MTDH was remarkably elevated in ccRCC tissues, especially in metastatic ccRCC tissues, compared with normal kidney tissues and correlated with advanced clinicopathological features and poor prognosis. MTDH activated ERK signaling and EMT, thus promoting the migration and invasion of ccRCC cells. The interaction between MTDH and SND1 at the protein level was confirmed using immunoprecipitation and immunofluorescence. Based on the analysis of datasets from GEO and TCGA, SND1 was remarkably increased in ccRCC, especially in metastatic ccRCC, and associated with advanced clinicopathological features and poor prognosis. Knockdown of SND1 mainly abolished the migration and invasion of ccRCC cells by blocking MTDH-mediated ERK and EMT signaling activation.

Conclusion: These results revealed that MTDH may be a prognostic metastatic biomarker of ccRCC that promotes ccRCC metastasis by activating SND1-mediated the ERK and EMT signaling pathways. MTDH may serve as an anti-tumor therapeutic target that can be applied for the clinical treatment of metastatic ccRCC.

Methods: MTDH/SND1 mRNA expression in clear cell renal cell carcinoma (ccRCC) was comprehensively estimated by analysis of GEO-ccRCC and TCGA-KIRC datasets with R software and packages. MTDH protein expression was assessed in a total of 111 ccRCC patients from Peking University First Hospital by immunohistochemistry (IHC). In vitro migration and invasion assays were carried out, and an in vivo metastatic mouse model was developed to investigate the biological functions of MTDH in ccRCC cells. Correlation analysis, immunoprecipitation, western blotting and immunofluorescence were applied to explore the molecular mechanisms of MTDH in ccRCC.

## INTRODUCTION

Clear cell renal cell carcinoma (ccRCC), the most common and lethal type of RCC (renal cell carcinoma), is one of the most common malignancies and exhibits high incidence and mortality rates [1]. Although the majority of patients with localized ccRCC have slow-growing, nonfatal tumors, some patients have disease recurrence and metastasis after complete surgical resection [2, 3]. Recurrence and metastasis are the principal causes of renal cell carcinoma-associated mortality [4]. Although molecular targeted drugs such as sunitinib and temsirolimus have been approved to treat patients with advanced metastatic ccRCC, the therapeutic effect of these drugs is limited to a short time interval, and the overall survival rate remains frustrating [2, 5, 6]. Therefore, exploring the molecular mechanisms of key steps in the metastatic progression of ccRCC is becoming essential.

MTDH, also called AEG1 and LYRIC, was shown to be involved in the carcinogenesis and progression of various types of tumors, including breast cancer [7], glioma [8, 9], hepatocellular carcinoma [10] and squamous cell carcinoma [11], suggesting that MTDH is a prognostic molecular biomarker and therapeutic target [4]. Many previous studies illustrated that MTDH can enhance tumor progression and metastasis through activating several classic cancer-promoting signaling pathways, such as epithelial-mesenchymal transition (EMT) [12, 13] and the NF-κB [14–16] and MAPK [17] signaling pathways. Furthermore, overexpression of MTDH is associated with chemotherapeutic resistance against several anticancer drugs, such as 5-fluorouracil [18], doxorubicin [19], paclitaxel [20, 21] and cisplatin [22]. Previous studies of ccRCC [18, 23, 24] suggested that increased MTDH expression is correlated with higher clinical staging, a more advanced grade and shorter patient survival, and knockdown of MTDH inhibited growth and induced apoptosis. The role and mechanism of MTDH in the metastatic progression of ccRCC remain unclear. Therefore, this study aimed to detect the expression of MTDH in ccRCC and further explore the role and mechanism of MTDH in the metastatic progression of ccRCC. In this study, we revealed that MTDH is remarkably increased in ccRCC, especially in metastatic ccRCC, and correlated with poor prognostic features. MTDH promoted metastasis of clear cell renal cell carcinoma by activating the SND1-mediated ERK and EMT signaling pathways.

## RESULTS

### The upregulation of MTDH is correlated with poor prognostic features

**Figure 1:**
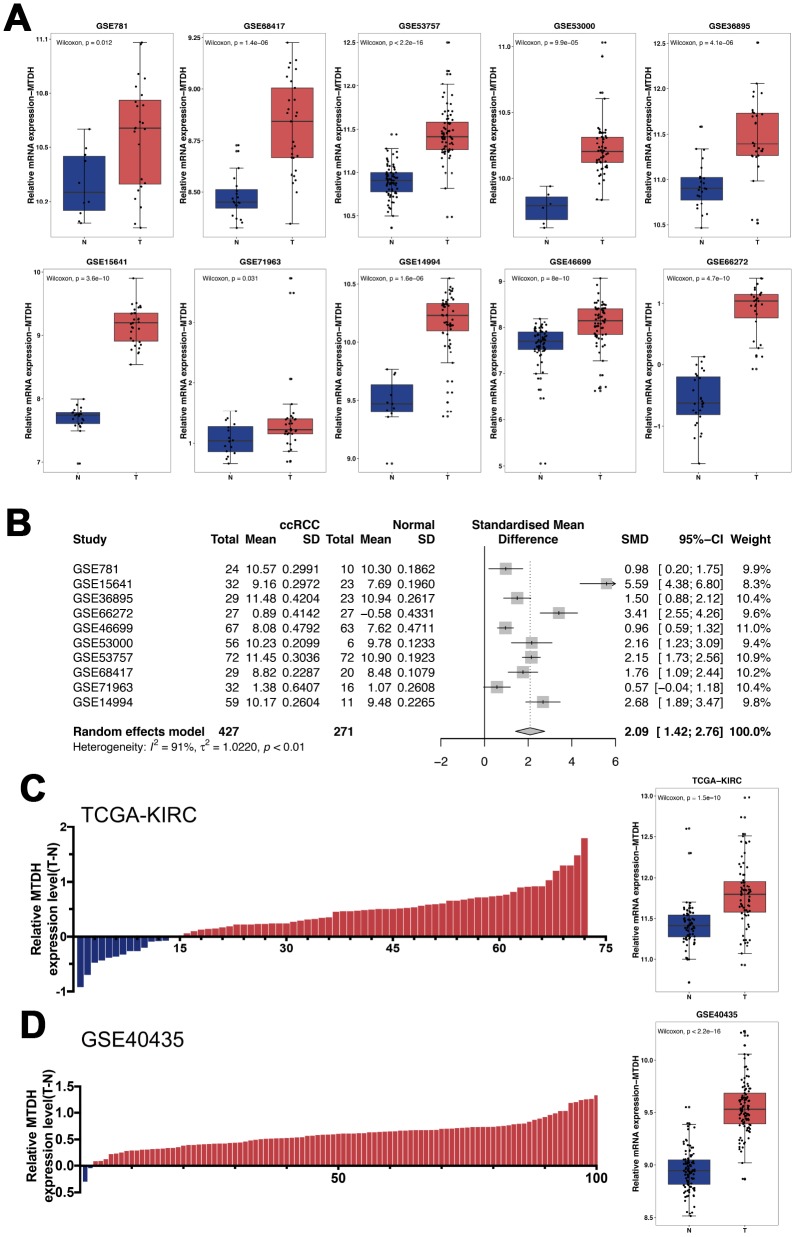
**The relative mRNA expression levels of MTDH in ccRCC patients.** (**A**) Based on GEO datasets, the mRNA expression of MTDH was significantly up-regulated in ccRCC tissues compared with normal kidney tissue in all 10 ccRCC GEO datasets. (**B**) The result of meta-analysis revealed that MTDH mRNA expression was still obviously increased in the union dataset. (**C**, **D**) Compared with matched paracancerous normal kidney tissues, MTDH mRNA expression was increased in 80.6%(58/72) and 98.0%(99/101) of ccRCC tissues in TCGA dataset and GSE40435, respectively.

To determine the mRNA expression of MTDH, RNA-seq and microarray data from ccRCC samples in datasets from The Cancer Genome Atlas (TCGA) and Gene Expression Omnibus (GEO) were analyzed. Among all 10 ccRCC GEO datasets, the mRNA expression of MTDH was significantly upregulated in ccRCC tissues compared with normal kidney tissues (Figure 1A). Meta-analysis revealed that MTDH mRNA expression was still obviously increased in the union dataset (Figure 1B, p < 0.001). Compared with matched paracancerous normal kidney tissues, MTDH mRNA expression was increased in 80.6% (58/72) and 98.0% (99/101) of ccRCC tissues described in the TCGA (Figure 1C) and GSE40435 (Figure 1D) datasets, respectively.

**Figure 2:**
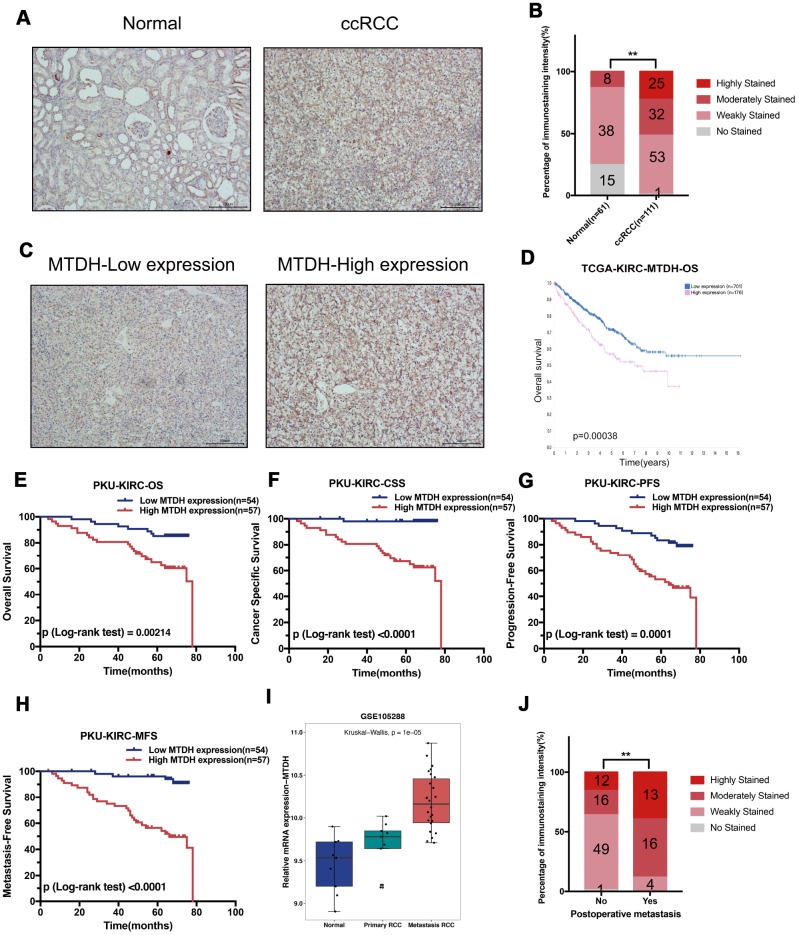
**Enhanced MTDH protein expression was associated with poor prognosis of ccRCC.** (**A**) The representative immunostaining of MTDH in normal kidney and ccRCC, (Scale bar, 200 μm). (**B**) Immunohistochemistry staining analysis of MTDH protein expression in 111 RCC tissues and 61 adjacent normal kidney tissues. (**C**) The representative immunostaining of high and low MTDH expression in ccRCC tissues was shown. (**D**) The high MTDH mRNA expression group had a poorer OS than the low MTDH group in TCGA-KIRC dataset based on The Human Protein Atlas website. (**E**) In 111 ccRCC patients from Peking University First Hospital, compared to those with lower expression levels, ccRCC patients with high MTDH protein expression levels had shorter OS. (**F**) In 111 ccRCC patients from Peking University First Hospital, compared to those with lower expression levels, ccRCC patients with high MTDH protein expression levels had shorter CSS. (**G**) In 111 ccRCC patients from Peking University First Hospital, compared to those with lower expression levels, ccRCC patients with high MTDH protein expression levels had shorter PFS. (**H**) In 111 ccRCC patients from Peking University First Hospital, compared to those with lower expression levels, ccRCC patients with high MTDH protein expression levels had shorter MFS. (**I**) MTDH mRNA expression in metastasis ccRCC is higher than the MTDH mRNA expression in normal kidney tissues and primary ccRCC in GSE105288 dataset. (**J**) Compared to those without postsurgical metastasis, ccRCC patients with postsurgical metastasis had higher MTDH protein expression levels in PKU-KIRC dataset.

To explore the protein expression of MTDH, 111 ccRCC samples included in the Peking University First Hospital-KIRC dataset (PKU-KIRC) were analyzed by immunohistochemistry. Representative immunostaining images for MTDH in normal kidney tissues and ccRCC tissues are shown in Figure 2A. The protein expression of MTDH was significantly enhanced in the ccRCC tissues compared with normal tissues (Figure 2B). Representative immunostaining images showing low (no staining and weak staining) and high (moderate staining and strong staining) MTDH expression in ccRCC tissues are shown in Figure 2C. We further analyzed the associations between MTDH immunostaining intensity with clinicopathological parameters. The results showed that MTDH protein expression was strongly associated with the maximum diameter of tumor (MDT), histologic grade, and pathologic T stage (Supplementary Figure 1A, Table 1). Collectively, these results indicate that elevated MTDH is significantly correlated with advanced clinicopathological features in ccRCC.

**Table 1:** Correlation between MTDH immunostaining intensity and clinicopathological features in 111 ccRCC patients from Peking University First Hospital.

**Clinicopathological features**		**MTDH expression**		**p**
		**Low**	**High**	
Age	<60	31	30	0.613
	≥60	23	27	
Gender	Female	22	18	0.315
	Male	32	39	
Maximum diameter of tumors	≤5cm	41	14	**<0.001**
	>5cm	13	43	
Histologic grade	G1	16	4	**<0.001**
	G2	36	37	
	G3	2	16	
Laterality	Left	19	26	0.166
	Right	34	28	
	Both	0	2	
pathologic T	T1-T2	54	22	**<0.001**
	T3-T4	0	35	
pathologic N	N0	54	55	0.5
	N1-2	0	2	
pathologic M	M0	54	56	1
	M1	0	1	

To explore the potential prognostic significance of MTDH, we evaluated the TCGA-KIRC dataset using the Human Protein Atlas website (https://www.proteinatlas.org/). As shown in Figure 2D, the group with high MTDH mRNA expression had a poorer overall survival (OS) than the group with low MTDH expression (p=0.00038). In the PKU-KIRC dataset, immunostaining confirmed that ccRCC patients with high MTDH protein expression levels had a shorter OS (Figure 2E, p=0.00214) and CSS (Figure 2F, p<0.0001). Moreover, ccRCC patients with high MTDH protein expression levels experienced recurrent or metastatic tumors earlier after radical nephrectomy (Figure 2G: PFS, p=0.0001 and Figure 2H: MFS, p<0.0001). Univariate Cox regression analyses indicated that increased maximum diameter of tumor values, histologic grade, laterality, pathologic T stage, pathologic N stage, pathologic M stage and MTDH levels were all related to poorer cancer-specific survival (CSS) and metastasis-free survival (MFS) in ccRCC patients. Multivariate Cox regression analysis indicated that MTDH is an independent prognostic factor for both CSS and MFS (Tables 2 and 3). In addition, both univariate and multivariate analyses of the PKU-KIRC dataset showed that ccRCC patients expressing high levels of MTDH had a shorter overall survival (OS) and progression-free survival (PFS) than patients with low MTDH expression (Supplementary Tables 3 and 4). Moreover, analysis of the GSE105288 dataset indicated that MTDH mRNA expression was higher in metastatic ccRCC tissues than in normal kidney tissues and primary ccRCC tissues (Figure 2I). In the PKU-KIRC dataset, MTDH protein expression was elevated in ccRCC patients with postsurgical metastasis (Figure 2J). Overall, these results demonstrate that increased MTDH expression is an independent predictive factor that may be involved in the metastatic progression of ccRCC.

**Table 2:** Uni- and multi-variate Cox regression of MTDH protein expression for cancer-specific survival (CSS) in 111 ccRCC from Peking University First Hospital.

**Clinicopathological features**	**Univariate analysis**		**Multivariate analysis**	
	**HR (95% CI)**	**p**	**HR (95% CI)**	**p**
Age(≥60 vs <60)	1.994(0.851,4.675)	0.112267		
Gender(Male vs Female)	0.817(0.333,2.004)	0.658755		
Maximum diameter of tumors (>5cm vs ≤5cm)	4.007(1.473,10.903)	**0.006574**		
Histologic grade (G3 vs G2 vs G1)	3.020(1.426,6.395)	**0.003883**		
Laterality(Left vs Right vs Both)	0.349(0.146,0.835)	**0.018**		
pT(T3/4 vs T1/2)	3.365(1.447,7.828)	**0.004845**		
pN(N1/2 vs N0)	21.165(4.106,109.098)	**<0.001**	17.903(2.983,107.441)	**0.001603**
pM(M1 vs M0)	54.498(4.942,601.024)	**0.001097**	90.945(5.200,1590.565)	**0.002007**
MTDH (High vs Low)	24.47093.286,182.200)	**0.001799**	20.072(2.666,151.095)	**0.003588**

**Table 3:** Uni- and multi-variate Cox regression of MTDH protein expression for metastasis-free survival(MFS) in 111 ccRCC from Peking University First Hospital.

**Clinicopathological features**	**Univariate analysis**		**Multivariate analysis**	
	**HR (95% CI)**	**p**	**HR (95% CI)**	**p**
Age(≥60 vs <60)	2.143(1.056,4.349)	**0.034704**	2.632(1.229,5.634)	**0.012733**
Gender(Male vs Female)	0.650(0.301,1.406)	**0.273776**		
Maximum diameter of tumors (>5cm vs ≤5cm)	3.321(1.531,7.205)	**0.002386**		
Histologic grade(G3 vs G2 vs G1)	3.991(2.121,7.509)	**<0.001**	2.019(1.050,3.882)	**0.035097**
Laterality(Left vs Right vs Both)	0.598(0.302,1.184)	0.140127		
pT(T3/4 vs T1/2)	4.862(2.386,9.907)	**<0.001**		
pN(N1/2 vs N0)	27.671(5.277,145.106)	**<0.001**	7.584(1.267,45.386)	**0.026453**
pM(M1 vs M0)	108.499(6.786,1734.639)	**<0.001**	99.904(5.333,1871.533)	**0.002073**
MTDH expression(High vs Low)	8.977(3.140,25.663)	**<0.001**	7.471(2.515,22.198)	**<0.001**

### MTDH promotes the migration and invasion of ccRCC cells

**Figure 3:**
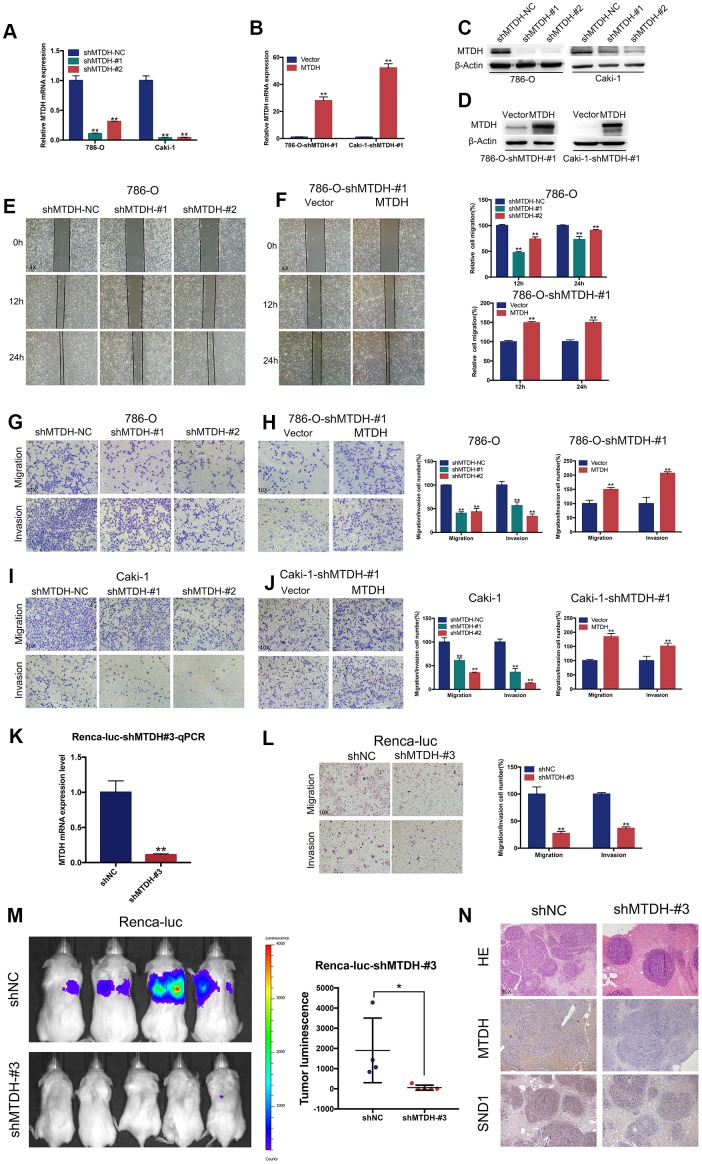
**MTDH promotes cell migration and invasion of ccRCC cells.** (**A**–**D**) RT-qPCR and western blot analyses of ccRCC cells infected with a lentivirus-mediated MTDH-overexpressing vector or MTDH shRNAs. (**E**–**F**) Wound-healing assay. Representative images of wound-induced cell migration by the 786-O-shMTDH, 786-O-#1-MTDH and control cells(4x). (**G**–**J**) Representative images of transwell migration and invasion assay of MTDH-knockdown cells and MTDH-overexpressed cells(10x). (**K**) RT-qPCR analyses of Renca-luc cells infected with a lentivirus-mediated MTDH shRNAs. (**L**) Representative images of transwell migration and invasion assay of Renca-luc-shMTDH#3(10x). (**M**) Tail vein-injected Renca-luc metastasis model. Representative IVIS images of mice injected mouse MTDH-silenced or control cells and analysis of tumor luminescence representing lung metastasis measured on day 21. Five mice per group (Renca-luc-shNC ccRCC cells failed in tail vein injection in one mice.) (**N**) Lung metastasis was confirmed by H&E and IHC- MTDH staining(10x).

To assess the ability of MTDH to promote metastasis, we carried out a series of functional assays using the relevant ccRCC cell lines. Then, ccRCC cell lines (786-O and Caki-1) stably overexpressing MTDH were established, and the ccRCC cell lines were transfected with shRNAs specifically targeting MTDH (Figure 3A–3D). To determine the role of MTDH in cell migration, a wound healing assay was conducted in 786-shMTDH and 786-shMTDH-#1-MTDH cells. Compared with the migration of control cells, the migratory capability was decreased in 786-shMTDH cells (Figure 3E) and increased in 786-shMTDH-#1-MTDH cells (Figure 3F). Transwell migration assays in two ccRCC cell lines also confirmed the above-mentioned results (Figure 3G–3J). Similarly, the invasion capability was determined using Transwell invasion assays. The invasion capability was significantly reduced in MTDH knockdown cells and enhanced in MTDH-overexpressing cells (Figure 3G–3J). To further confirm that MTDH promotes cell metastasis, a tail vein injection metastasis model was developed using luciferase-labeled Renca (Renca-Luc) cells. Renca-luc cells stably expressing shRNAs specifically targeting MTDH were established (Figure 3K, 3N). The migratory and invasive capabilities were significantly reduced in Renca-luc-shMTDH cells compared with control cells (Figure 3L). A significant reduction in tumor metastasis in the MTDH knockdown group compared with the control group was identified (Figure 3M). Overall, the above results demonstrated that MTDH promotes the migration and invasion of ccRCC cells both in vitro and in vivo.

### MTDH enhances metastasis by promoting ERK signaling and EMT

**Figure 4:**
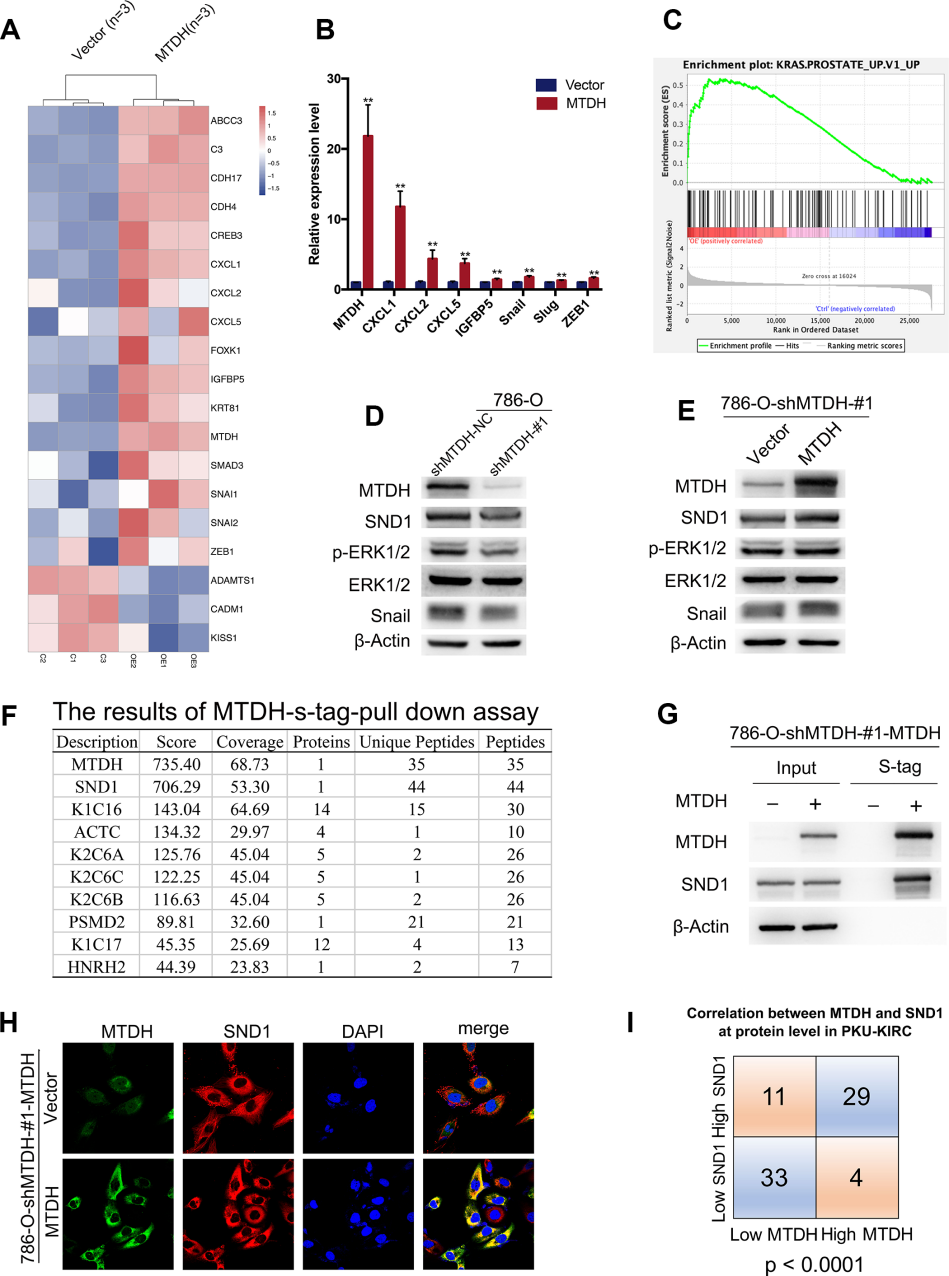
**MTDH promotes metastasis by activating ERK signaling and EMT.** (**A**) Heatmap representation of differentially expressed genes identified by RNA-Seq between 786-O-shMTDH-#1-MTDH cells (n = 3) and 786-O-shMTDH-#1-vector Control cells (n = 3). (**B**) Validation of differentially expressed genes by RT-qPCR. Comparison of mRNA expression of genes in pathways of cancer (CXCL1/2/5 and IGFBP5) and genes in EMT-related pathway (Snail, Slug and ZEB1) between 786-O—shMTDH-#1-MTDH cells and 786-O—shMTDH-#1-vector Control cells. All data are shown as means ± SD. (**C**) Based on our own RNA sequencing data, genes influenced by MTDH overexpression were mostly enriched in pathways involved with KRAS signaling using Gene Set Enrichment Analysis (GSEA) pathway analysis. (**D**) Silencing MTDH reduced the protein expression of p-ERK1/2, Snail and SND1 in ccRCC cells. (**E**) Overexpressing MTDH increased the protein expression of p-ERK1/2, Snail and SND1 in ccRCC cells. (**F**) The result of mass spectrometry analysis of s-tag pull down assay confirm the interaction between MTDH and SND1 at the protein level. (**G**) MTDH and SND1 were co-localized, mainly in the cytoplasm. (**H**) The result of immunoprecipitation revealed that MTDH binds to SND1 at the protein level. (**I**) The correlation of MTDH and SND1in PKU-KIRC dataset was statistically analyzed (P<0.0001).

To explore the potential mechanism by which MTDH promotes metastasis, RNA sequencing and bioinformatics were applied to analyze the potential pathways involved in MTDH. mRNA sequencing of 786-O-shMTDH-#1-MTDH and 786-O-shMTDH-#1-vector Control cells was conducted, which revealed that many genes were differentially expressed when MTDH was overexpressed. These differentially expressed genes could be used to separate 786-O-shMTDH-#1-MTDH cells from control cells (Figure 4A). The genes screened by RNA-seq were validated by RT-qPCR. CXCL family genes (*CXCL1, CXCL2, CXCL5*) and EMT-related genes (*Snail, Slug, ZEB1*) were significantly upregulated in 786-O-shMTDH-#1-MTDH cells compared to control cells (Figure 4B). Based on our own RNA sequencing data obtained using gene set enrichment analysis (GSEA) pathway analysis, genes influenced by MTDH overexpression were mostly enriched in pathways involved with KRAS signaling (Figure 4C). These results were also confirmed using TCGA-KIRC RNA sequencing data (Supplementary Figure 1B). Next, the above findings were confirmed at the protein level using western blotting. p-ERK1/2 and the EMT inducer snail were dramatically reduced in 786-O-shMTDH-#1 cells (Figure 4D), but upregulated in 786-O-shMTDH-#1-MTDH cells (Figure 4E). Compared with control cells, 786-O cells with over-expression of MTDH showed loss of adherent phenotype with decreased intercellular contact and Induction of fibroblast like state (Supplementary Figure 1G). Furthermore, Kyoto Encyclopedia of Genes and Genomes (KEGG) pathway analysis revealed that genes influenced by MTDH overexpression were mostly enriched in pathways involved in PI3K-AKT signaling, prostate cancer, extracellular matrix (ECM)-receptor interactions and focal adhesion (Supplementary Figure 1C). Pathways analysis of TCGA-KIRC RNA sequencing data by GSEA also demonstrated that differentially expressed genes due to MTDH upregulation were mostly enriched in the PI3K-AKT signaling pathway (Supplementary Figure 1D). Moreover, MTDH also increased the expression of p-NF-κB and p-p38, executing its function in promoting cancer (Supplementary Figure 1E).

### MTDH promotes metastasis largely by enhancing SND1-mediated EMT and ERK signaling

To identify MTDH-interacting proteins that potentially affect its function in ccRCC cells, S-tag pulldown assays and mass spectrometry analysis of 786-O-shMTDH-#1-MTDH cells were conducted. Mass spectrometry analysis of proteins pulled down through the S-tag pulldown assay (Figure 4F) confirm the interaction between MTDH and SND1 at the protein level, which has been demonstrated in previous studies [7, 26–28]. However, many new MTDH-interacting proteins that may be interesting for understanding the function of MTDH were identified (Figure 4F). Next, we further analyzed the mRNA expression patterns of MTDH and SND1 in ccRCC tissues. Among the eight GEO datasets and the TCGA-KIRC dataset (Supplementary Figure 1E), a significant positive correlation between MTDH and SND1 was identified (Spearman’s correlation r: 0.361-0.766, all p < 0.01). The protein expression patterns of MTDH and SND1 in ccRCC tissues were assessed by IHC. As shown in Figure 4I, a strong positive correlation between MTDH and SND1 at the protein level was revealed. Previous studies have revealed that MTDH promotes tumorigenesis under oncogenic/stress conditions by interacting with and stabilizing Staphylococcal nuclease domain-containing 1 (SND1) [7, 29]. To confirm the interaction between MTDH and SND1 at the protein level, immunofluorescence and immunoprecipitation experiments were performed in ccRCC cells infected with a lentivirus-mediated MTDH overexpression vector. The immunofluorescence results indicated that MTDH and SND1 were colocalized, mainly in the cytoplasm (Figure 4H). The interaction between MTDH and SND1 at the protein level was confirmed using immunoprecipitation (Figure 4G), which suggests that MTDH promotes metastasis via SND1.

**Figure 5:**
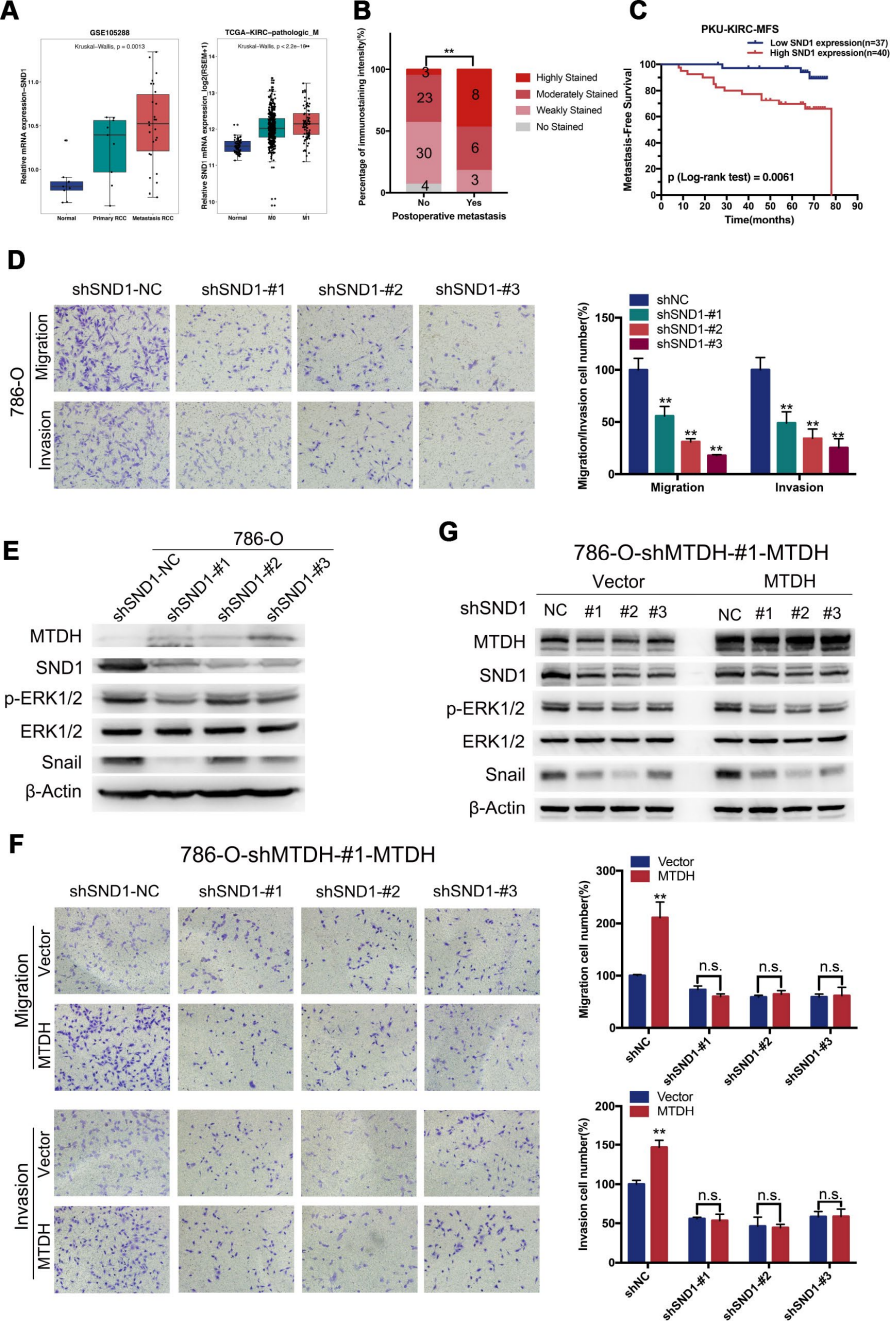
**MTDH promotes metastasis largely by enhancing SND1-mediated EMT and ERK signaling.** (**A**) SND1 mRNA expression was increased in ccRCC tissues in both TCGA dataset and GSE105288, especially in metastatic ccRCC. (**B**) Compared to those without postsurgical metastasis, ccRCC patients with postsurgical metastasis had higher SND1 protein expression levels in PKU-KIRC dataset. (**C**) The ccRCC patients with high SND1 protein expression levels had shorter MFS than those with lower SND1 expression levels (p = 0.0061). (**D**) Migration and invasive capabilities were attenuated in 786-O-SND1-knockdown cells(20x). (**E**) Silencing SND1 reduced the protein expression of p-ERK1/2, Snail in 786-O cells. (**F**) knockdown of SND1 could abolish promoting migration and invasive capabilities of ccRCC cells induced by overexpressing MTDH(20x). (**G**) Silencing SND1 could attenuate increased expression of snail and p-ERK protein induced by overexpressing MTDH.

SND1 has been reported to contribute to tumor progression and metastasis in various types of tumors, including glioma [30], breast cancer [31–33] and hepatocellular carcinoma [34]. However, the expression and molecular function of SND1 in ccRCC remain completely unclear. To explore the expression of SND1 in ccRCC, RNA-seq and microarray data from ccRCC samples in the TCGA and GEO datasets were analyzed. Among all 12 ccRCC GEO datasets, SND1 mRNA expression was significantly increased in ccRCC tissues compared with normal kidney tissues (Supplementary Figure 2A). Meta-analysis revealed that SND1 mRNA expression was still obviously enhanced in the union dataset (Supplementary Figure 2B, p<0.0001). SND1 mRNA expression was also significantly upregulated in ccRCC tissues compared with normal kidney tissues (Figure 5A). Compared with that in matched paracancerous normal kidney tissues, SND1 mRNA expression was increased in ccRCC tissues described in the TCGA-KIRC dataset (Supplementary Figure 2C). We further analyzed the association of SND1 expression with clinicopathological parameters based on the TCGA-KIRC dataset. SND1 expression was strongly associated with histologic grade, pathologic T stage, pathologic N stage, pathologic M stage and pathologic stage (Supplementary Figure 2F, Supplementary Table 5). To explore the potential prognostic significance of SND1, we evaluated the TCGA-KIRC dataset using the Human Protein Atlas website (https://www.proteinatlas.org/). As shown in Supplementary Figure 2D and 2E, the group with high SND1 mRNA expression had a poorer OS and shorter relapse-free survival (RFS) than the group with low SND1 mRNA expression (OS: p = 0.014, RFS: p = 0.0151). Furthermore, SND1 mRNA expression in metastatic ccRCC was higher than SND1 mRNA expression in normal kidney tissues and primary ccRCC tissues when the GSE105288 and TCGA-KIRC datasets were analyzed (Figure 5A). Representative immunostaining images showing low (no staining and weak staining) and high (moderate staining and strong staining) MTDH expression in ccRCC tissues are shown in Supplementary Figure 1H. In the PKU-KIRC dataset, SND1 protein expression was elevated in ccRCC patients with postsurgical metastasis (Figure 5B), and ccRCC patients with high SND1 protein expression levels had a shorter MFS than those with low SND1 protein expression levels (Figure 5C, p = 0.0061). These results indicated that SND1 may contribute to the metastatic progression of ccRCC.

To explore the potential function and mechanism of SND1 in promoting metastasis, a 786-O ccRCC cell line that stably expressed shRNAs specifically targeting SND1 (786-O-shSND1 cells) was established. Compared with control cells, 786-O-shSND1 cells showed dramatically weakened migration and invasion capabilities (Figure 5D). Consistent with the involvement of SND1 in cancer metastasis via ERK signaling and EMT revealed in previous studies, we found that p-ERK1/2 and the EMT inducer snail were significantly reduced in 786-O-shSND1 cells using western blotting (Figure 5E). The abovementioned results offer powerful support for a functional correlation between MTDH and SND1 during tumor progression and metastasis in ccRCC. To explore whether MTDH promotes metastasis in an SND1-dependent manner, the migration and invasive capabilities of 786-O-shMTDH-#1-MTDH ccRCC cells infected with a lentivirus-mediated shSND1 vector were determined. As shown in Figure 5F, knockdown of SND1 abolished the increased migration and invasion capabilities of ccRCC cells promoted by MTDH overexpression. Silencing SND1 largely abolished the increased expression of the p-ERK1/2 and snail proteins induced by MTDH overexpression (Figure 5G). Overall, the above results revealed that MTDH promotes metastasis mainly by enhancing SND1-mediated ERK signaling and EMT.

## DISCUSSION

MTDH, a metastasis-related gene with significant prognostic potential and therapeutic value in various types of tumors, is involved in multiple processes [35], including survival, angiogenesis and metastasis. In ccRCC, only the correlation between the MTDH expression level and clinicopathological characteristics and the ability of MTDH knockdown to inhibit cell growth were investigated in previous studies. Staphylococcal nuclease domain-containing 1 (SND1), a transcriptional coactivator, was identified as an oncoprotein involved in carcinogenesis and the progression of various types of tumors [30, 32, 33, 36]. Currently, the expression and function of SND1 in ccRCC remain poorly understood. In this study, both MTDH and SND1 were remarkably increased in ccRCC, especially in metastatic ccRCC, and associated with advanced clinicopathological features and poor prognosis. The interaction between MTDH and SND1 at the protein level was confirmed using immunoprecipitation and immunofluorescence. MTDH promoted the metastasis of clear cell renal cell carcinoma by activating SND1-mediated ERK signaling and epithelial-mesenchymal transition.

Based on bioinformatics analysis of the TCGA-KIRC and GEO-ccRCC datasets, both MTDH and SND1 were significantly increased in ccRCC, especially in metastatic ccRCC, and correlated with advanced clinicopathological features. An elevated MTDH protein level is an important prognostic factor independent of other clinicopathological features. We further confirmed the ability of MTDH and SND1 to promote migration and invasion in ccRCC cells using Transwell assays. To better understand the oncogenic function of MTDH in ccRCC, RNA sequencing and bioinformatics were used to analyze the potential pathways in which MTDH is involved. EMT and KRAS signaling were shown to be induced by the overexpression of MTDH using bioinformatics. The KRAS signaling pathway, a well-known cancer-related signal transduction pathway, is involved in tumor metastasis and progression [37, 38]. *ERK* is a key gene in the KRAS signaling pathway [39]. Our results demonstrated that the overexpression of both MTDH and SND1 promoted the migration and invasion of ccRCC cells by activating ERK and EMT. Similar results were obtained in some previous studies [11, 40]. Moreover, a significant positive correlation between MTDH and SND1 was found in the eight GEO datasets and the TCGA-KIRC dataset, and the interaction between MTDH and SND1 at the protein level was confirmed using immunoprecipitation and immunofluorescence, which suggested that their functions and molecular mechanisms involved in tumor progression are closely connected. To determine whether MTDH promotes metastasis in an SND1-dependent manner, the migration and invasion capabilities of 786-O-shMTDH-#1-MTDH ccRCC cells infected with a lentivirus-mediated shSND1 vector were determined. As expected, the results suggested that MTDH promotes ccRCC cell migration and invasion largely by enhancing SND1-mediated ERK signaling and epithelial-mesenchymal transition. As for how MTDH regulates SND1 to promote metastasis, there is no clear explanation yet. Only one previous study has mentioned that MTDH can promote tumorigenesis under oncogenic/stress conditions by interacting with and stabilizing Staphylococcal nuclease domain-containing 1 (SND1) [7]. The same result was also observed in our study (Figure 5D, 5E). As for how MTDH stabilizes SND1 and how MTDH promotes metastasis through SND1, further research is needed in the future.

The current standard treatment for metastatic ccRCC is a combination of surgical resection to remove the primary tumor and targeted drugs to eliminate metastatic tumors [4]. However, relapsed cancers are largely resistant to targeted drugs, and most cannot be resected by surgery. Previous studies revealed the vital roles of MTDH in the metastasis and chemoresistance of cancer cells, uncovering the potential of MTDH as an antitumor therapeutic target [35]. Our results suggested that MTDH promotes ccRCC cell metastasis largely in an SND1-dependent manner. Although no selective MTDH inhibitors have been found, our research provides new ideas and emphasizes the use of selective SND1 inhibitors and inhibitors that block the binding of MTDH to SND1.

Furthermore, KEGG pathway analysis revealed that the genes influenced by MTDH overexpression are mostly enriched in the PI3K-AKT signaling pathway. Previous studies have confirmed that the PI3K/AKT signaling pathway is involved in MTDH-mediated metastasis and cell survival [11, 41]. By triggering the PI3K/AKT signaling pathway, MTDH regulates the downstream expression of genes encoding metastasis-associated proteins and apoptosis-associated proteins (Bad, p21 and p27) and contributes to the induction of a malignant cell phenotype [41]. The mechanism by which MTDH regulates these downstream genes and the identification of functional partners of MTDH are important topics for future research.

## CONCLUSIONS

Overall, these results suggest that MTDH is a novel independent predictive factor based on its adverse prognostic effects and a metastatic driver that enhances SND1-mediated EMT and ERK signaling. Exploring the molecular mechanism of MTDH and SND1 in the metastatic progression of ccRCC should be emphasized in future studies. MTDH, a vital driver of metastasis, may serve as a therapeutic target that can be applied to the clinical treatment of metastatic ccRCC.

## MATERIALS AND METHODS

### Bioinformatics data mining

All ccRCC gene microarray profiling data with a sample size greater than 20 (GSE781, GSE15641, GSE16449, GSE36895, GSE46699, GSE53000, GSE53757, GSE68417, GSE71963, GSE14994, GSE40435, GSE66272, GSE105288) were included in this study and obtained from the Gene Expression Omnibus (GEO, https://www.ncbi.nlm.nih.gov/geo/). TCGA-KIRC-MTDH/SND1 gene expression data and clinical data were downloaded from UCSC Xena (http://xena.ucsc.edu/). R software and packages were applied to preprocess the RNA sequencing and microarray data.

### Patients and clinical materials

Between January 2007 and December 2010, a total of 111 ccRCC patients who had undergone radical nephrectomy without preoperative therapy were enrolled in this study. This study was approved by the Biomedical Research Ethics Committee of Peking University First Hospital, and written informed consent was obtained from all enrolled patients. According to the 2011 Union for International Cancer Control TNM classification of malignant tumors, all tissue samples were confirmed by pathology as clear cell-type RCC. The nuclear grade was determined by the Fuhrman nuclear grading system. All animal studies were conducted according to the guidelines of the Institutional Animal Care and Use Committee of Peking University First Hospital.

### Cell culture and transfection

The 786-O, Caki-1 and Renca ccRCC cell lines were obtained from the Institute of Cell Research, Chinese Academy of Sciences, Shanghai, China. Cell lines were cultured according to conditions specified by the provider. The sequences of the related MTDH/SND1-shRNA and a negative control shRNA were designed, and the shRNAs were chemically synthesized and inserted into the pLKO.1 vector. The MTDH sequence was generated by PCR and inserted into the pLVX vector with a C-terminal S-tag label. Lentiviruses were produced in HEK-293T cells using a three-vector system consisting of targeting vector: viral packaging vector (psPAX2): viral envelope vector (pMD2G) at a 4:3:1 ratio. The ccRCC cells were selected with puromycin (2 μg/mL) and blasticidin (3 μg/mL).

### RNA extraction and real-time quantitative PCR (RT-qPCR)

According to the manufacturer’s instructions, the total RNA was extracted from tissue samples and the transfected cells by using TRIzol reagent (Invitrogen; Thermo Fisher Scientific, Inc.). cDNA was generated by using reverse transcription (TansGEN, Beijing, China). RT-qPCR was performed using the ABI PRISM 7000 fluorescent quantitative PCR system (Applied Biosystems, Foster City, CA, USA) according to the manufacturer’s instructions, and expression levels were normalized to those of TUBA. Detailed information on the primer sequences included in this study is shown in Supplementary Table 1.

### Immunohistochemistry (IHC) and Western blot analysis

Immunohistochemistry (IHC) experiments and IHC scoring were carried out as previously described according to previous protocols [25]. Protein lysates were prepared by homogenization in 1% NP-40 containing 1 mM PMSF, and samples containing 20 μg of protein were separated by SDS-PAGE. The immunoreactive bands were visualized using an ImmobilonTM Western kit (Millipore, Billerica, MA) using the Syngene G:BOX imaging system (Frederick, USA). According to the manufacturer’s instructions, western blot assays were carried out with antibodies diluted in PBS plus Tween-20 (PBST). Information on all primary antibodies used is listed in Supplementary Table 2.

### Wound healing assay

Wounds were marked and photographed with a microscope (Leica DM IL, Leica Microsystems, Germany) equipped with a digital camera (Leica DFC300FX) at 0, 12 and 24 h after scratching with a sterile 200 μL pipette. The scratched area was measured at three different positions.

### Transwell migration and invasion assay

For Transwell migration assays, 10000 cells were plated into the upper chambers of a Transwell apparatus (24-well insert, 8 μm pore size, Corning) with 200 μL of serum-free RPMI-1640. The lower chambers were filled with 700 μL of RPMI-1640 containing 10% fetal bovine serum. Twenty-four hours later, the adherent cells on the lower surface were stained with 0.5% crystal violet in methanol for 30 minutes.

For invasion assays, 1000 cells were seeded on a Transwell apparatus (24-well insert, 8 μm pore size, Corning) coated with Matrigel (diluted 1:8 in PBS, product #354234, Corning, Inc., NY, USA). The culture conditions were the same as those described for the Transwell migration assay. After 24 hours, the adherent cells on the lower surface were stained with 0.5% crystal violet in methanol for 30 minutes. Cells on the lower surface were photographed with a microscope (Leica DM IL, Leica Microsystems) equipped with a digital camera (Leica DFC300FX) and counted.

### Metastatic mouse model development by tail vein injection

Renca-luc cells stably expressing luciferase were used in this experiment. Approximately 5×10^5^ Renca-luc-shMTDH-NC and Renca-luc-shMTDH-#3 cells were injected into the lateral tail vein of each 5-week-old male NSG mouse (SPF (Beijing) Biotechnology Co., Ltd., China). Three weeks after injection, the mice were anesthetized with isoflurane (Yipin Pharmaceutical, China), and D-luciferin sodium salt (Biovision, USA) was then injected intraperitoneally according to the manufacturer’s instructions. Metastatic lesions were visualized using the Xenogen IVIS in vivo imaging system (Perkin Elmer, MA, USA).

### Immunofluorescence

Cells from the different groups were seeded on glass slides at 24 h prior to the experiment and grown. After fixation with 4% paraformaldehyde–PBS for 15 min, the cells were washed once with PBS and permeabilized with 0.5% Triton X-100. The cells were then stained with the appropriate primary antibodies at 4 °C for 12 h. Nuclei were stained with 0.2 mg/mL DAPI.

### Immunoprecipitation

Cells were collected and lysed in 1% NP-40 with 1 mM PMSF. Whole-cell lysates (2 mg) were precleared with 20 μL of S-tag beads and incubated at 4 °C for 12 hours on a rocking platform. The immune complexes were subjected to SDS-PAGE and analyzed by immunoblotting.

### S-tag pull down assay and mass spectrometry analysis

Protein lysates were prepared by cells homogenization in 1% NP40 buffer containing PMSF and proteinase inhibitor cocktail (Roche). 0.5-1mg protein lysates were incubated with S-protein beads (Novagen) at 4°C for 2h. After centrifuge and washed three times by 1% NP40 buffer, the precipitated complex was boiled with protein loading buffer and separated by SDS-PAGE. The gel was stained by silver stainning kit (Beyotime). The precipitated complex subsequently analyzed by mass spectrum. Mass spectrometry analysis was conducted using an AB SCIEX MALDI TOF-TOF 5800 Analyzer.

### RNA-seq data analysis

RNA-seq experiments were conducted by Novogene (Beijing, China). Briefly, the total RNA was isolated from 786-O-shMTDH-#1-vector Control cells and 786-O-shMTDH-#1-MTDH cells cells using TRIzol reagent (Invitrogen) and treated with RNase-free DNase I (New England Biolabs, MA, USA) to remove any contaminating genomic DNA. RNA extraction was performed using Dynabeads oligo(dT) (Invitrogen Dynal). Double-stranded complementary DNA was synthesized from 1 μg of total RNA using Superscript II reverse transcriptase (Invitrogen) and random hexamer primers. *Escherichia coli* RNase H (New England Biolabs) was added to remove RNA complementary to the cDNA. The cDNA was then fragmented by nebulization, and the standard Illumina protocol was followed thereafter to create mRNA-seq libraries. The libraries were sequenced on an Illumina HiSeq 2000 platform. Sequencing reads were aligned to the human genome (hg19) using the TopHat program (version 2.1.1) set to the default parameters. Total read counts for each protein-coding gene were extracted using HTSeq (version 0.6.0) and then loaded into the R package DESeq2 to calculate the differentially expressed genes with a cut-off fold change of ≥1.5 and an FDR < 0.05. Gene expression levels were calculated as fragments per kilobase of transcript per million mapped reads (FPKM). Gene set enrichment analysis (GSEA) was applied for gene functional annotation.

### Statistical analyses

All statistical tests were performed using SPSS version 23.0 software (SPSS, Inc., Chicago, IL, USA) and R version 3.5.2. All experimental data were analyzed by Student’s t-test, the χ2 test or the nonparametric test. Survival curves were plotted using the Kaplan-Meier method with log-rank tests. Univariable and multivariable Cox (forward method: Wald test) regression analyses were applied to assess the prognostic relevance between clinicopathological and immunohistochemical data. Variables with a value of p < 0.05 in univariate analysis were included in subsequent multivariate analysis on the basis of the Cox proportional hazards model. Differences for which p<0.05 were considered statistically significant (*p < 0.05, **p < 0.01, n.s. > 0.05).

### Ethics approval

This study was approved by Biomedical Research Ethics Committee of Peking University First Hospital and written informed consents were obtained before any operation to patients. Consent for publication. The authors confirmed that written consent have be obtained from the patients to publish this manuscript.

## Supplementary Material

Supplementary Figures

Supplementary Tables
