# Correction for: MTDH promotes metastasis of clear cell renal cell carcinoma by activating SND1-mediated ERK signaling and epithelial-mesenchymal transition

**DOI:** 10.18632/aging.205603

**Published:** 2024-04-15

**Authors:** Anbang He, Shiming He, Cong Huang, Zhicong Chen, Yucai Wu, Yanqing Gong, Xuesong Li, Liqun Zhou

**Affiliations:** 1 Department of Urology, Peking University First Hospital, Beijing 100034, China; 2 Institute of Urology, Peking University, Beijing 100034, China; 3 National Urological Cancer Center, Beijing 100034, China

**Keywords:** clear cell renal cell carcinoma, metastasis, MTDH, SND1, EMT, ERK

**This article has been corrected:** It was found that there is the overlap between the images showing migration and invasion of Caki-1-shMTDH-KD1-MTDH cells in **Figure 3J.** The authors confirmed that the image of transwell invasion of Caki-1-shMTDH-KD1-MTDH cells was misplaced during the figure layout. They provided the original images of the transwell assays and corrected **Figure 3J** with the proper image obtained from the original data. The authors also would like to clarify the use of the same western blot images in **Figures 3D** and **4E**, depicting MTDH and β-actin expression in 786-O-shMTDH-#1 cells. The purpose was to demonstrate the efficacy of MTDH overexpression in lentivirus-infected cells, which is now stated in the legend to **Figure 4E**. These corrections do not change the results or conclusions of the article. The authors apologize for any confusion or inconvenience possibly caused.

The corrected version of **Figure 4** legend and **Figure 3** are provided below.

**Figure 4. MTDH promotes metastasis by activating ERK signaling and EMT. **(**A**) Heatmap representation of differentially expressed genes identified by RNA-Seq between 786-O-shMTDH-#1-MTDH cells (n = 3) and 786-O-shMTDH-#1-vector Control cells (n = 3).  (**B**) Validation of differentially expressed genes by RT-qPCR. Comparison of mRNA expression of genes in pathways of cancer (CXCL1/2/5 and IGFBP5) and genes in EMT-related pathway (Snail, Slug and ZEB1) between 786-O--shMTDH-#1-MTDH cells and 786-O--shMTDH-#1-vector Control cells. All data are shown as means ± SD. (**C**) Based on our own RNA sequencing data, genes influenced by MTDH overexpression were mostly enriched in pathways involved with KRAS signaling using Gene Set Enrichment Analysis (GSEA) pathway analysis. (**D**) Silencing MTDH reduced the protein expression of p-ERK1/2, Snail and SND1 in ccRCC cells. (**E**) Overexpressing MTDH increased the protein expression of p-ERK1/2, Snail and SND1 in ccRCC cells. (786-O-shMTDH-#1 cells MTDH and b-actin images were used in Figure 3D to demonstrate MTDH overexpression efficacy after lentiviral infection). (**F**) The result of mass spectrometry analysis of s-tag pull down assay confirm the interaction between MTDH and SND1 at the protein level. (**G**) MTDH and SND1 were co-localized, mainly in the cytoplasm. (**H**) The result of immunoprecipitation revealed that MTDH binds to SND1 at the protein level. (**I**) The correlation of MTDH and SND1in PKU-KIRC dataset was statistically analyzed (P<0.0001).

**Figure 3:**
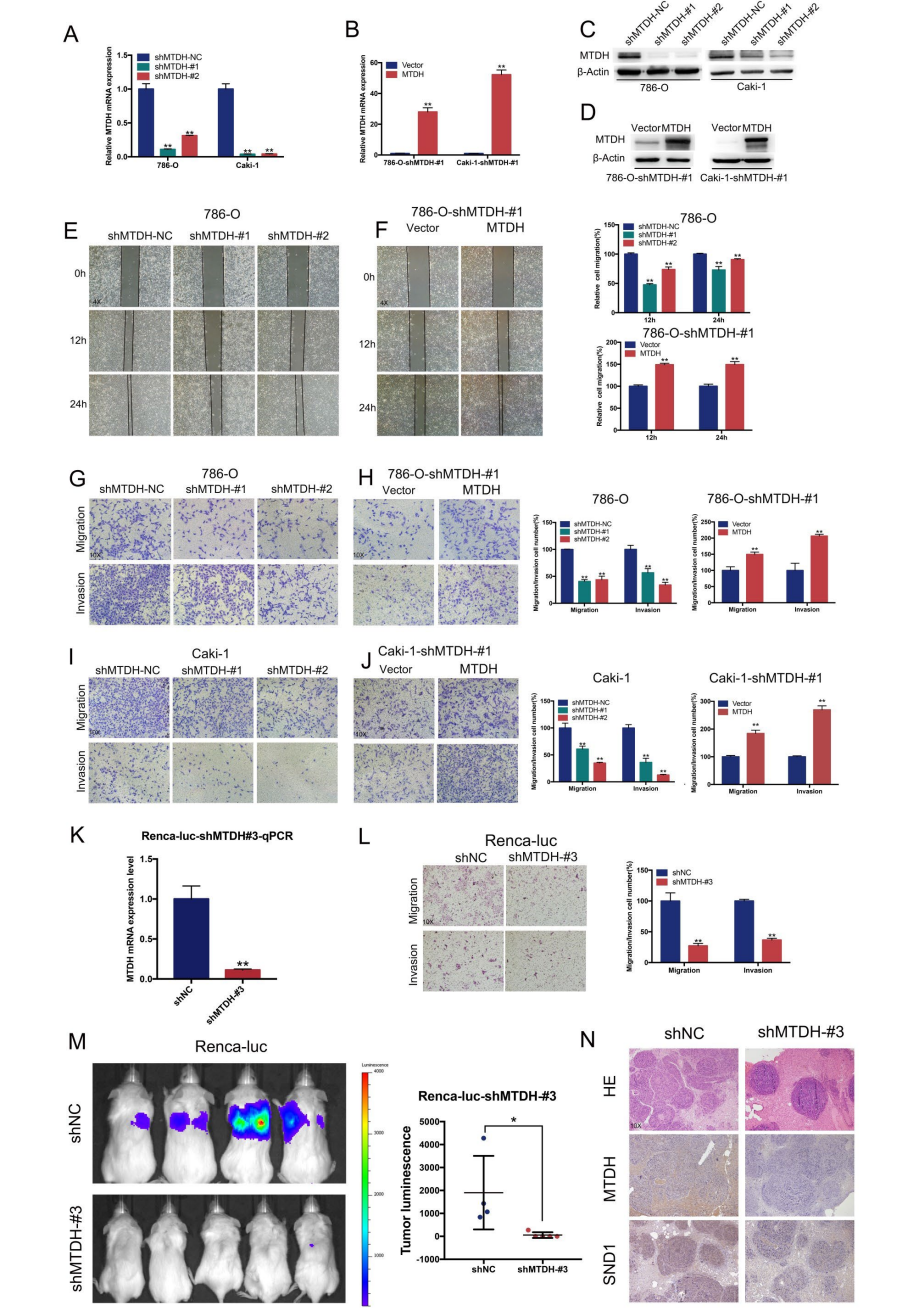
**MTDH promotes cell migration and invasion of ccRCC cells.** (**A**–**D**) RT-qPCR and western blot analyses of ccRCC cells infected with a lentivirus-mediated MTDH-overexpressing vector (**D**) or MTDH shRNAs (**C**). (**E**–**F**) Wound-healing assay. Representative images of wound-induced cell migration by the 786-O-shMTDH, 786-O-#1-MTDH and control cells(4x). (**G**–**J**) Representative images of transwell migration and invasion assay of MTDH-knockdown cells and MTDH-overexpressed cells(10x). (**K**) RT-qPCR analyses of Renca-luc cells infected with a lentivirus-mediated MTDH shRNAs. (**L**) Representative images of transwell migration and invasion assay of Renca-luc-shMTDH#3(10x). (**M**) Tail vein-injected Renca-luc metastasis model. Representative IVIS images of mice injected mouse MTDH-silenced or control cells and analysis of tumor luminescence representing lung metastasis measured on day 21. Five mice per group (Renca-luc-shNC ccRCC cells failed in tail vein injection in one mice.) (**N**) Lung metastasis was confirmed by H&E and IHC- MTDH staining(10x).

