# Supplementary material for: MTDH promotes metastasis of clear cell renal cell carcinoma by activating SND1-mediated ERK signaling and epithelial-mesenchymal transition
Source: Aging (Albany NY). 2020 Jan 24;12(2):1465–87. doi: 10.18632/aging.102694 (PMC7053596; doi:10.18632/aging.102694)
Supplement: Supplementary Figures [file aging-12-102694-s002..pdf]

SUPPLEMENTARY FIGURES

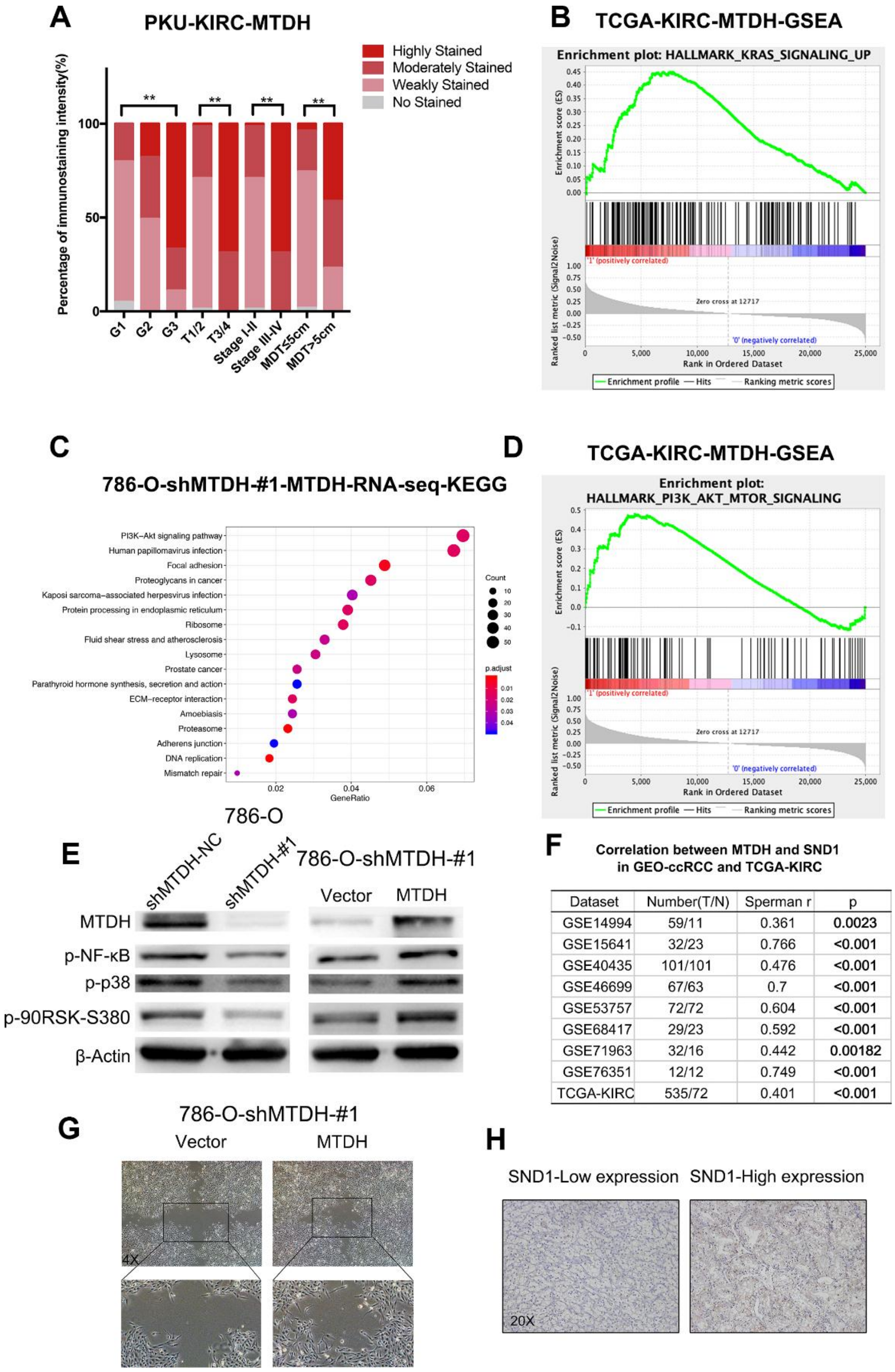

Dataset	Number(T/N)	Sperman r	p
GSE14994	59/11	0.361	**0.0023**
GSE15641	32/23	0.766	**<0.001**
GSE40435	101/101	0.476	**<0.001**
GSE46699	67/63	0.7	**<0.001**
GSE53757	72/72	0.604	**<0.001**
GSE68417	29/23	0.592	**<0.001**
GSE71963	32/16	0.442	**0.00182**
GSE76351	12/12	0.749	**<0.001**
TCGA-KIRC	535/72	0.401	**<0.001**

Supplementary Figure 1. Potential mechanism of tumor oncogenic function of MTDH in ccRCC. (**A**) Percentage distribution of MTDH IHC intensity in ccRCC with various clinicopathological features. histologic grade, pathologic T, pathologic stage and MDT. (**B**) Based on TCGA-KIRC RNA sequencing data, genes influenced by MTDH overexpression were mostly enriched in pathways involved with KRAS signaling

using Gene Set Enrichment Analysis (GSEA) pathway analysis. (**C**) Statistics of KEGG pathway enrichment. The y-axis corresponds to KEGG Pathway, and the x-axis shows the GeneRatio. The color of the dot represents adjusted p value (padj), and the size of the dot represents the number of differentially expressed genes mapped to the reference pathways. (**D**) Based on TCGA-KIRC RNA sequencing data, genes influenced by MTDH overexpression were enriched in pathways involved with PI3K-AKT signaling using Gene Set Enrichment Analysis (GSEA) pathway analysis. (**E**) Western blot assay of p-NF-κB, p-P38 and p-P90RSK expression in MTDH-knockdown 780-O cells and MTDH-overexpressed cells, comparing with control cells. (**F**) A significant positive correlation between MTDH and SND1 was identified (Spearman's correlation r: 0.361-0.766, all p <0.01) in the eight GEO datasets and TCGA-KIRC dataset. (**G**) compared with control cells, 786-O cells with over-expression of MTDH showed loss of adherent phenotype with decreased intercellular contact and Induction of fibroblast like state. (**H**) The representative immunostaining of high and low SND1 expression in ccRCC tissues was shown.

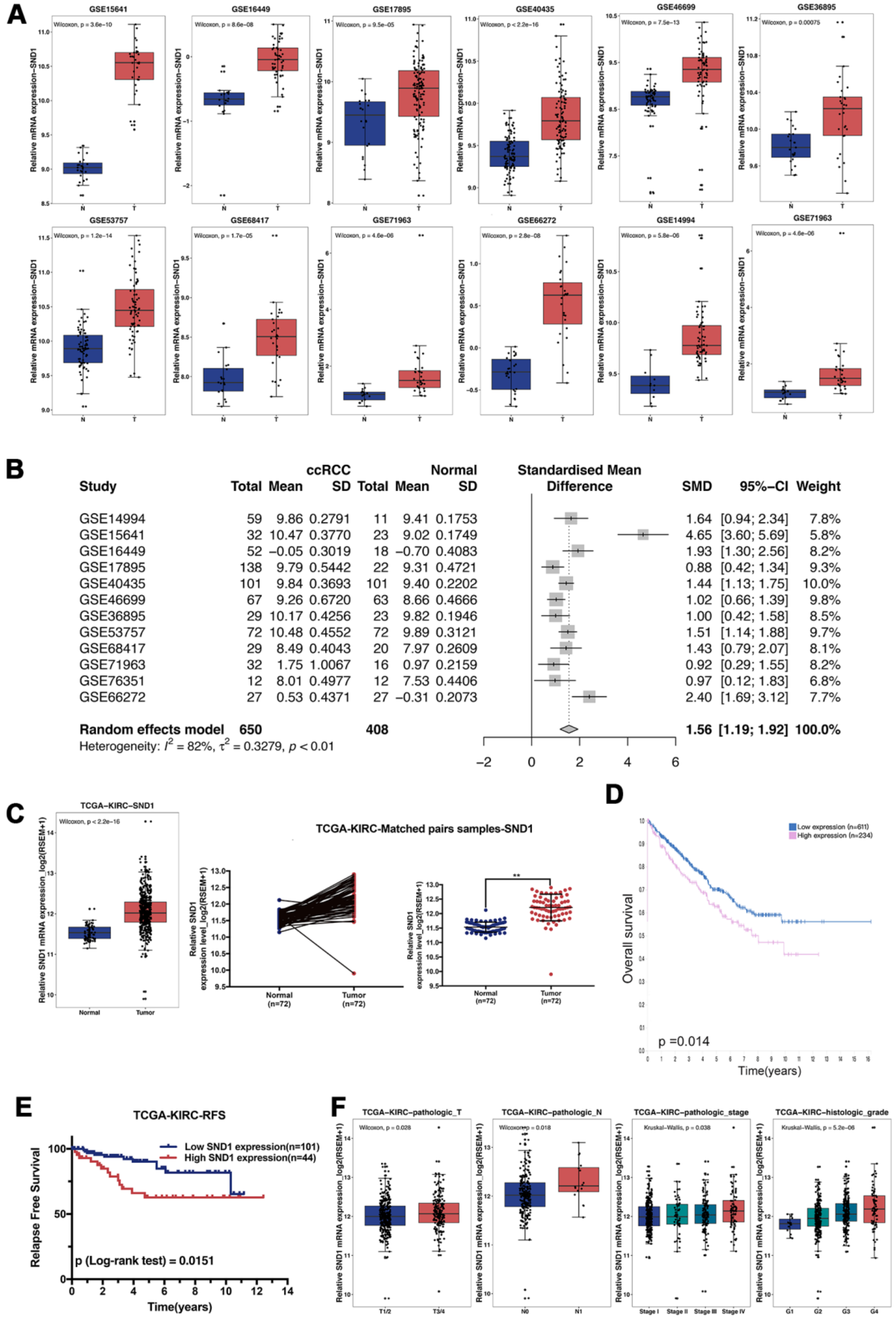

Supplementary Figure 2. The relative SND1 mRNA expression in ccRCC. (**A**) The mRNA expression of SND1 was significantly increased in ccRCC tissues compared with normal kidney tissue in all 12 ccRCC GEO datasets. (**B**) The result of meta-analysis revealed that SND1 mRNA expression was still obviously upregulated in the GEO union dataset. (**C**) Compared with matched paracancerous normal kidney tissues, SND1 mRNA expression was increased in TCGA-KIRC dataset. (**D**) The high SND1 mRNA expression group had a poorer OS than the low SND1 group in TCGA-KIRC dataset based on The Human Protein Atlas website. (**E**) In TCGA-KIRC dataset, ccRCC patients with high SND1 expression levels had shorter RFS (p_log-rank test=0.0151). (**F**) The SND1 mRNA expression is upregulated in ccRCC patients with higher pathologic T, pathologic N, pathologic stage and histologic grade in TCGA-KIRC dataset.
