# Supplementary material for: MTDH promotes metastasis of clear cell renal cell carcinoma by activating SND1-mediated ERK signaling and epithelial-mesenchymal transition
Source: Aging (Albany NY). 2020 Jan 24;12(2):1465–87. doi: 10.18632/aging.102694 (PMC7053596; doi:10.18632/aging.102694)
Supplement: Supplementary Tables [file aging-12-102694-s001..pdf]

SUPPLEMENTARY TABLES

Supplementary Table 1. Characteristics of the corresponding qPCR primers and shRNAs included in the context.

Genes	Forward primer	Reverse primer
MTDH	AAATAGCCAGCCTATCAAGACTC	TTCAGACTTGGTCTGTGAAGGAG
CXCL1	CTTCAGGAACAGCCACCAGT	TCCTGCATCCCCCATAGTTA
CXCL2	GCTTGTCTCAACCCCGCATC	TGGATTTGCCATTTTTCAGCATCTT
CXCL5	AGCTGCGTTGCGTTTGTTTAC	TGGCGAACACTTGCAGATTAC
Snail	CCCCAATCGGAAGCCTAACTAC	GAGCCTTTCCCACTGTCCTCAT
Slug	ATTCGGACCCACACATTACCTTG	GATGAGCCCTCAGATTTGACCT
ZEB1	TAAGCAAACGATTCTGATTCCC	CCTCTACATTTGATACTCCTTCTG
TUBA	GATTCCTTCAACACCTTCTTCAG	GTGCGAACTTCATCAATGAC

shRNA	Sequence
ShMTDH-#1	CATCTGCAGCCGAGGAATAAA
ShMTDH-#2	TGAAGACTGGCCTTATTAATG
Mouse-shMTDH-#3	GCTTTGATCAAATGTATAATC
shSND1-#1	TCCACCGTGTTGCAGATATAT
shSND1-#2	GACAGCGTAGTTCGGGATATC
shSND1-#3	TGTCCTAGTGGAGGGAGTAAT
ShRNA-NC	CCGAATTACTCCTAGAACCGC

Supplementary Table 2. Characteristics of the corresponding antibodies included in the context.

Protein	Assay	Antibody	Origin	Dilution	Incubation period
MTDH	IHC<br>WB<br>IF	13860-1-AP proteintech	Rabbit	1/10000<br>1/2000<br>1/500	overnight, 4°C
SND1	WB<br>IHC<br>IF	60265-1-Ig proteintech	Mouse	1/1000<br>1:20000<br>1/200	overnight, 4°C
Snail	WB	9728T CST	Rabbit	1/1000	overnight, 4°C
p-ERK1/2-S217/221	WB	9911T CST	Rabbit	1/1000	overnight, 4°C
ERK1/2	WB	16443-1-AP proteintech	Rabbit	1/1000	overnight, 4°C
p-NF-κB-S536	WB	ab76302 abcam	Rabbit	1/1000	overnight, 4°C
p-P38-T180/Y182	WB	Ab195049 abcam	Rabbit	1/1000	overnight, 4°C
p-90RSK-S380	WB	9911T CST	Rabbit	1/1000	overnight, 4°C
β-actin	WB	TA-09, ZSGB Bio	Mouse	1/2000	overnight, 4°C
goat anti-rabbit IgG-HRP	WB	sc-2030, Santa Cruz	Goat	1/5000	1 hour, RT
goat anti-mouse	WB	sc-516102, Santa Cruz	Goat	1/5000	1 hour, RT

IgG-HRP					
goat anti-rabbit Alexa Fluor® 488	IF	ab150077, abcam	Goat	1/200	1 hour, RT
goat anti-mouse Alexa Fluor® 647	IF	ab150075, abcam	Goat	1/200	1 hour, RT

Abbreviations: IHC, immunohistochemistry; IF, immunofluorescence; WB, Western blot; RT, room temperature. CST, Cell signaling Technology

Supplementary Table 3. Uni- and multi-variate Cox regression of MTDH protein expression for overall survival (OS) in 111 ccRCC from Peking University First Hospital.

Clinicopathological features	Univariate analysis		Multivariate analysis	
	HR (95% CI)	p	HR (95% CI)	p
Age(≥60 vs <60)	2.086(1.003,4.337)	**0.048935**		
Gender(Male vs Female)	0.745(0.341,1.627)	0.460226		
Maximum diameter of tumors (>5cm vs ≤5cm)	2.873(1.310,6.299)	**0.008414**		
Histologic grade(G3 vs G2 vs G1)	2.213(1.161,4.217)	**0.015798**		
Laterality(Left vs Right vs Both)	0.300(0.140,0.640)	**0.001875**	0.365(0.168,0.793)	**0.010943**
pT(T3/4 vs T1/2)	1.956(0.937,4.080)	0.073896		
pN(N1/2 vs N0)	17.620(3.556,87.304)	**<0.001**	11.016(1.950,62.215)	**0.006602**
pM(M1 vs M0)	54.498(4.942,601.024)	**0.001097**	67.389(3.911,1161.209)	**0.003745**
MTDH expression(High vs Low)	3.318(1.473,7.478)	**0.00381**	2.609(1.128,6.031)	**0.024923**

Supplementary Table 4. Uni- and multi-variate Cox regression of MTDH protein expression for progression-free survival (PFS) in 111 ccRCC from Peking University First Hospital.

Clinicopathological features	Univariate analysis		Multivariate analysis	
	HR (95% CI)	p	HR (95% CI)	p
Age(≥60 vs <60)	2.281(1.216,4.279)	**0.010187**	3.122(1.587,6.144)	**<0.001**
Gender(Male vs Female)	0.683(0.348,1.339)	0.266791		
Maximum diameter of tumors (>5cm vs ≤5cm)	2.864(1.478,5.551)	**0.001826**	2.576(1.176,5.641)	**0.017967**
Histologic grade(G3 vs G2 vs G1)	2.960(1.691,5.179)	**<0.001**		
Laterality(Left vs Right vs Both)	0.518(0.283,0.948)	**0.033049**		
pT(T3/4 vs T1/2)	3.245(1.747,6.027)	**<0.001**	2.059(0.988,4.290)	0.053772
pN(N1/2 vs N0)	19.906(4.079,97.158)	**<0.001**	6.537(1.239,34.483)	**0.026904**
pM(M1 vs M0)	54.498(4.942,601.024)	**0.001097**	67.113(5.499,819.144)	**<0.001**
MTDH expression(High vs Low)	3.550(1.773,7.108)	**<0.001**		

Supplementary Table 5. Correlation between SND1 mRNA expression and clinicopathological features of ccRCC patients in TCGA-KIRC dataset.

Clinicopathological features		SND1 mRNA expression		p
		**Low**	**High**	
Age	<60	125	118	0.514402
	≥60	138	146	
Gender	Female	104	81	0.033077
	Male	159	183	
Histologic grade	G1/2	141	98	**<0.001**
	G3/4	117	163	
pT	T1-T2	181	157	**0.025214**
	T3-T4	82	107	
pN	N0	120	119	**0.014768**
	N1-2	3	13	
pM	M0	212	205	**0.015371**
	M1	28	50	
